# A Review of Chitosan-Based Materials for Biomedical, Food, and Water Treatment Applications

**DOI:** 10.3390/ma17235770

**Published:** 2024-11-25

**Authors:** Dan Chicea, Alexandra Nicolae-Maranciuc

**Affiliations:** 1 Research Center for Complex Physical Systems, Faculty of Sciences, Lucian Blaga University of Sibiu, 550012 Sibiu, Romania; 2 Institute for Interdisciplinary Studies and Research (ISCI), Lucian Blaga University of Sibiu, 550024 Sibiu, Romania

**Keywords:** chitosan, biomaterial, biodegradable, antibacterial activity, tissue engineering, hydrogel, bioprinting, drug delivery systems, food packaging, water treatment

## Abstract

Chitosan, a natural biopolymer with excellent biocompatibility, biodegradability, and modifiable structure, has broad applications in regenerative medicine, tissue engineering, food packaging, and environmental technology. Its abundance, solubility in acidic solutions, and capacity for chemical modification make it highly adaptable for creating specialized derivatives with enhanced properties. Recent advances have demonstrated chitosan’s efficacy in composite systems for tissue regeneration, drug delivery, and antimicrobial applications. This review examines chitosan’s unique properties, with a focus on its antibacterial activity as influenced by factors like pH, concentration, molecular weight, and deacetylation degree. Additionally, chitosan’s potential as a sustainable, non-toxic material for eco-friendly packaging and water treatment is explored, highlighting the growing interest in chitosan composites with other polymers and metallic nanoparticles for enhanced biomedical and environmental applications.

## 1. Introduction

Polysaccharides are a category of macromolecular polymers involved in various biological systems due to their abundance in nature. Part of the carbohydrate polymer class, polysaccharides can be extracted from many natural sources such as plants, animals, algae, or microorganisms [1,2]. As biopolymers, cellulose, starch, or chitosan are among the most suitable matrices for tissue reconstruction or antitumoral/antimicrobial agents in biomedical applications [3,4,5]. Biomaterials based on natural biopolymers are an innovative alternative to classical synthetic materials since first, they are biocompatible with tissues and biological structure, and secondly, they sustain the environment, being biodegradable and obtained from renewable sources [6]. These biodegradable polymers, including chitosan, facilitate the patients’ conditions when they are used in surgical procedures for medical purposes, while they also offer an improvement in life quality regarding the environmental impact of the biodegradable plastic industry [7,8,9].

Chitosan, part of the natural biopolymers class, is used in the development of bioengineered medical devices and food packaging films since it has multiple benefits in improving human medical conditions. Tissue engineering, a field where polymers are the main components and where chitosan is used in various forms and ways, showed increased benefits while integrating nanomaterials for tissue function improvements together with a polymeric matrix [10]. The advantages based on high biocompatibility, biodegradability, and high flexibility offered by chitosan materials led to the development of biomaterials for skin injuries [11] and bone reconstruction [12] and for drug delivery therapies [13]. Its biological properties are completed by the superior properties of nanoparticles when a nanocomposite is designed. For instance, silver nanoparticles offer a high antibacterial effect [14,15], while gold nanoparticles are extremely engaged in biosensor detections [16]; lately, both have been integrated into various forms of chitosan biomaterials. Shinde et al. [17] addressed the worldwide multidrug-resistant bacteria problem by proposing a green system based on chitosan-coated silver nanoparticles as antimicrobial agents for medical applications. The results showed that bioconjugates based on chitosan and silver nanoparticles exhibit a stronger bactericidal effect on Gram-positive and Gram-negative strains compared to simple silver nanoparticles while also showing no cytotoxicity in the L-929 fibroblast cell line [17]. Badawy et al. [18] proved a strong antibacterial effect of nanocomposites based on chitosan and silver nanoparticles against *Escherichia coli* (*E. coli*) strain, also in the food packaging industry [18]. Nevertheless, our previous study showed that nanocomposites based on chitosan and chemically reduced silver nanoparticles can inhibit bacterial growth against *E. coli* and *Staphylococcus aureus* (*S. aureus*) strains [14]. Another study that shows the antifungal activity of chitosan was conducted by Ipinza-Concha et al. [19]. The authors obtained a bioconjugate based on chitosan and riboflavin as photoactive fungicides against *Penicillium digitatum*, a postharvest pathogen in fruits, as an alternative solution to synthetic fungicide products [19].

This review aims to offer information about chitosan, as part of the polysaccharide class, with implications in biomedical applications, especially as hydrogels for biodegradable and antibacterial applications. As a natural polymer, chitosan can overcome the problems encountered in the case of synthetic polymers by offering promising support for further regeneration processes. The development of composite polymers based on chitosan and various materials will also be approached in this work. The association of chitosan with nanoparticles and nanocomposites is a promising strategy for smart and efficient systems in bioengineering.

## 2. Chitosan: Extraction Process and Chemical Structure

### 2.1. Chitosan Extraction

Chitin, a natural component used in polymers science, is among the first polysaccharides identified, preceding even cellulose by about 30 years. The first clue about chitin was identified in 1811 when Henry Braconnot (1780–1855) extracted chitin from mushrooms [20]. Later, Professor C. Rouget discovered during an experiment that once an alkali treatment is applied to chitin, a new and different substance able to be dissolved in acids is obtained. This substance was named chitosan by Hoppe-Seiler in 1894 as the deacetylated result of chitin [21]. As a natural material with multiple properties and benefits, the interest in chitosan and chitosan-based materials has increased gradually until now.

**Figure 1:**
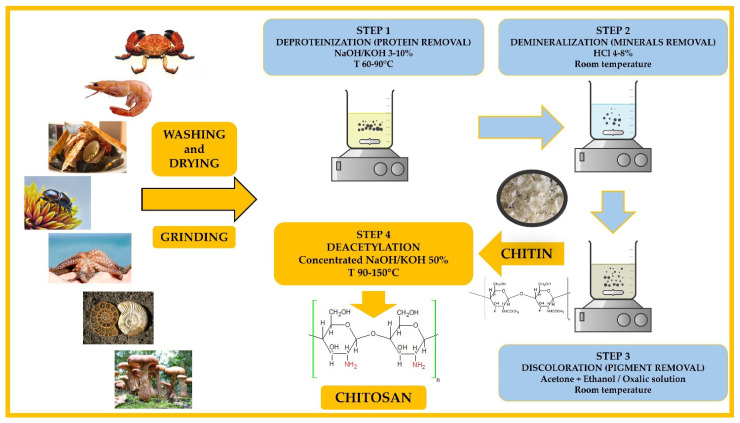
Chitosan extraction process from natural sources.

**Figure 2:**
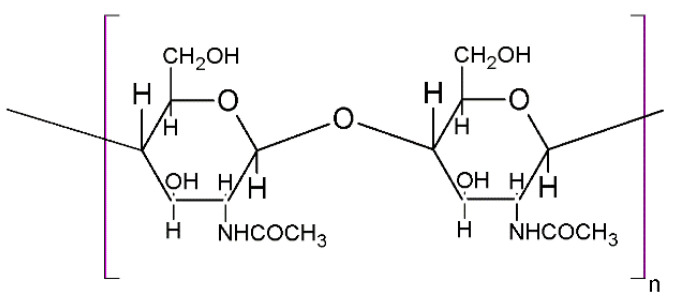
Chemical structure of chitin.

Terrestrial organisms, marine organisms, microorganisms, the cell walls of fungi, or crustacean shells [22] are examples of natural sources from which chitosan can be extracted and processed [23]. Chitosan is an extraordinarily abundant pseudo-natural cationic polymer found in high percentages in nature, yet less than cellulose. Chemically, as in the case of collagen, chitosan can be extracted under different conditions, leading to compounds with various chemical configurations and structures. Shrimp and crab crustaceans are the main sources of α-chitosan, the form with the best properties in bioengineering applications [24]. In the case of chitosan, its beneficial properties for human health led to the investigation of different strategies for its integration into biomaterials. During medical evolution, many treatments were used with the purpose of increasing the solubility of this polymer in aqueous media (e.g., deacetylation, carboxymethylation, sulfation) [25,26]. Chitosan is obtained, with the best yield, from chitin through a process that involves four classical stages, as described in Figure 1: deproteinization with a less concentrated NaOH solution, demineralization using HCl solutions, discoloration using acetone and ethanol to achieve chitin, and lately, deacetylation using strong NaOH solutions and high temperatures to achieve chitosan [27,28,29]. The deacetylation process, shown in Figure 2, is the most important and known process since it manages all the final solubility, crystallinity, and degradability properties of chitosan materials. Following several extraction steps based on an alkali treatment and high temperatures, a typical chitosan solution soluble in acidic media is obtained [30].

The parameter that guides this process is called the degree of deacetylation (DD) [24,31,32]. This degree of deacetylation shows the percentage of β-1,4-D-glucosamine remaining in the chitosan structure [33]. A reaction limit could be considered at 50% DD since chitosan solubility in acid is conditioned by a higher percentage than this limit [24,31]. However, depending on the parameters of the deacetylation, the obtained DD classifies chitosan as high deacetylated chitosan (70–99%) or low deacetylated chitosan (55–70%) [33,34,35]. The DD can commonly be measured through many techniques, such as nuclear magnetic resonance/infrared (IR)/ultraviolet (UV) spectrophotometry [36], enzymatic reactions [37], or chemical methods (e.g., titration) [38]. The final physical–chemical properties of chitosan are strongly influenced by the chemical structure; therefore, DD plays an important role in biomedical applications [25]. Gîjiu et al. [27] reported in a recent study an optimization of extraction steps for a chitosan solution with a high degree of deacetylation, starting from crab waste from the North Sea, Romania, as the main source. The process involved variations in HCl and NaOH concentrations, temperature, and a number of acidic treatments applied using a Taguchi method, in which many factors involved are analyzed in the process. The results showed that a high DD is obtained using NaOH 5% and a temperature of around 65 °C up to 75 °C, while HCl and the number of acidic treatments have a lower impact on the extraction process [27].

Temperature, chemical structure, concentration, solvent, and degree of deacetylation are factors that control the intrinsic properties of the final solution. Besides DD, another important parameter that can affect chitosan bio-functionality, swelling properties, or solubility in its final applications is the molecular weight (MW) [33,39]. MW depends on the number of monomeric units of the backbone. Chitosan can be found as a high-molecular-weight (MW > 700 kDa), medium-molecular-weight (150 < MW < 700 kDa), or low-molecular-weight (50 < MW < 150 kDa) substance or as an oligochitosan (MW < 50 kDa) [33]. Chromatography and viscometry measurements are the main methods for determining the molecular weight of chitosan [40]. The influence of MW and DD on chitosan physicochemical properties and final applications was investigated through many studies. For instance, the study by Mania et al. [41] showed that the antibacterial effect of chitosan xerogels with various MWs is different for *E. coli* and *S. aureus* strains. The bacterial inhibition was lower for a high MW chitosan with a mass of around 4000 kDa. The possible mechanism, according to authors, could be correlated to the aggregates formed by hydrogen interactions when such large molecules are used [41]. Joseph et al. [34] reviewed the applications of chitosan depending on the DD and MW. The review shows that a high deacetylation degree with high molecular weight is used in scaffolds and dressings in tissue engineering, drug delivery systems, and food packaging, while a high deacetylation degree with low molecular weight is suitable for wastewater treatment and metal reduction in environmental studies [34]. Carrera et al. [42] compared a chitosan with low DD/high MW and another sample with high DD/low MW obtained from biowaste to observe the modifications that appeared at the molecules’ surfaces and how the molecules were arranged depending on both parameters. The results of the average particle size of chitosan solutions showed a larger size for the sample with low DD and high MW, regardless of the concentration studied. Additionally, the study suggests that once the aggregate sizes are bigger and the MW increases, the surface activity increases, leading to the formation of adsorbed multilayers at the air/water surface [42]. In another paper, Román-Doval et al. [43] studied the antibacterial and antifungal activities of chitosan using various molecular weights in agriculture. The study showed that when using chitosan with low molecular weight, bacterial inhibition is stronger compared to chitosan with high molecular weight [43].

Chitosan is a non-toxic, semi-crystalline biopolymer, yet insoluble in water or some organic solvents. It is a hydrophilic polymer, also considered a weak base, with high solubility in acidic solutions and low mechanical strength [44]. Since solubility is a problem for materials synthesis, the chosen solvents are really important for obtaining strong biomaterials. The stability of chitosan also depends on the chemical factors of the environment. Its solubility can be influenced by the pH of the medium, one of the most important parameters in the manufacturing process. At pH 6.0 and lower, the amino -NH_2_- groups become protonated; thus, chitosan dissolves in strong acidic solutions [45].

### 2.2. Chitosan Chemical Structure and Chemical Derivatives

**Figure 3:**
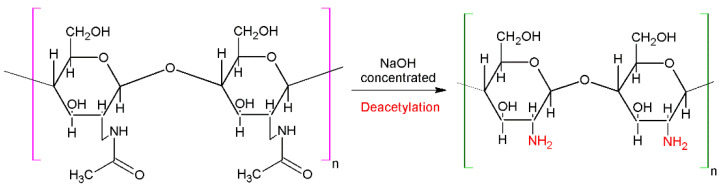
Deacetylation reaction for chitosan extraction.

Chitin, with the chemical structure described in Figure 2, and chitosan possess some similarities with cellulose structure. However, the main difference appears at C-2 in the cellulose structure, where the hydroxyl group is replaced with an acetamide group [46]. Chitosan, a partially deacetylated structure of chitin, is a linear polysaccharide composed of β-(1–4)-linked D-glucosamine and N-acetyl-D-glucosamine units randomly mixed [47]. The chemical reaction of chitosan extraction is based on the transformation of almost all the acetamide groups into primary amino groups [46] by a hydrolysis process, as can be observed in Figure 3. In almost all cases, some residues of acetamide groups are still found in the chitosan structure; however, due to the presence of these amino groups, chitosan is chemically more active [46,48].

**Figure 4:**
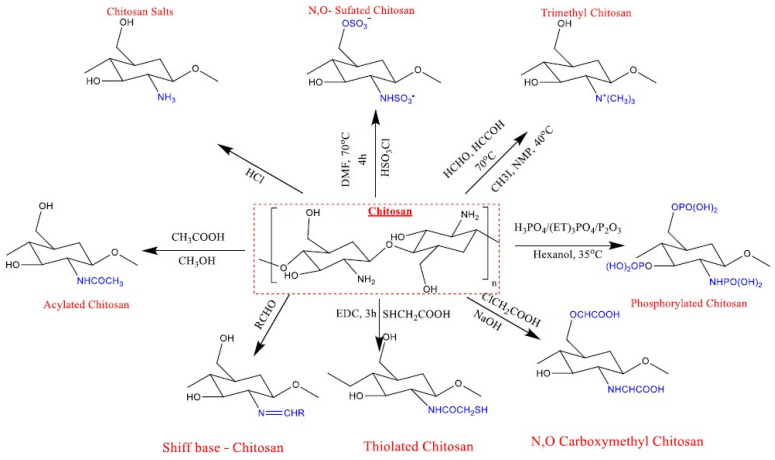
Chitosan modifications based on different chemical reactions. Reprinted from an open-source article [49].

Its reactivity allows various chemical modifications to strengthen some specific properties; therefore, many applications can be proposed based on its biocompatibility and biodegradability. In the chemical structure of chitosan, there are different types of reactive groups: amino groups, primary hydroxyl groups at C-3, and secondary hydroxy groups at C-6 carbon [49]. Their reactivity is stronger at the amino groups compared to the primary hydroxyl groups, which are also stronger compared to the last secondary hydroxyl groups [49,50]. The versatility offered by the chitosan molecular structure allows for chitosan functionalization at amino or hydroxyl groups with different active molecules [51] in medical applications, as can be observed in Figure 4, or its bonding to other materials, such as polymers or nanoparticles, for properties improvements. In a recent study, Wang et al. [52] proved that derivatives based on hydroxypropyl chitosan and quaternary ammonium salt of chitosan have a more powerful scavenging effect on 2,2-diphenyl-1-picryl hydrazyl radicals for an antioxidant effect compared to a chitosan sample [52].

## 3. Chitosan Properties Related to Its Biomedical Application

### 3.1. Biocompatibility

Chitosan attracts significant interest for medical and pharmaceutical applications due to its exceptional properties of biocompatibility, biodegradability, and non-toxicity, making it highly valuable in the biomedical field [53,54]. Biocompatibility is the main aspect related to biomedical applications since it does not induce modifications in tissues’ immune system responses. Its biocompatibility manages to not activate the body’s protection mechanisms and ensures compatibility with living cells; therefore, materials containing chitosan are more easily accepted [55]. Medical research has shown that chitosan exhibits a low level of toxicity while it is used as a biomaterial due to the support offered [56].

Chitosan also has unique properties, including hemostatic properties through red blood cell aggregation, platelet adhesion, and further aggregation [57]; antithrombogenic qualities; and the ability to form polyoxysalts, create films, and exhibit molecular adsorption capabilities. Studies have shown that chitosan is a biocompatible and biodegradable biopolymer with antimicrobial properties that enhance blood coagulation [58,59].

### 3.2. Biodegradability

Biodegradability is one of the most important properties of the natural polymers used in various types of applications. However, in the case of regenerative medicine, biodegradability is essential for materials developed to offer support while the tissue is healing. The process of biodegradation can be considered a mechanical one if the degradation occurs due to swelling, cracking, or dissolution or a chemical one if enzymatic processes occur [60,61]. Chitosan is degraded through enzymatic hydrolysis by an enzyme found in human tissues called lysozyme [62] or with the aid of lipase [63,64,65], the enzyme found in pancreatic fluid or saliva. Chitosan is primarily degraded by enzymes called chitinases and chitosanases, which are specifically designed to break down chitin and chitosan. Studies like [66,67] focus on using chitosan as a support material for immobilizing lipases rather than as a substrate for lipase degradation. While lipases may not directly degrade chitosan, they could potentially contribute to its breakdown indirectly by modifying the surface properties of chitosan or interacting with other components in a complex system, potentially facilitating degradation by other means. The biodegradation of chitosan releases biocompatible molecules [68], showing no toxicity in the area implanted or around it. This process is usually described in three steps: chitosan weight slowly decreases, the lysozyme, together with water molecules, permeates the internal structure of chitosan, and finally, the biopolymer is slowly degraded by the enzymes [60,69].

Chitosan can be catalyzed by enzymes in order for the molecule to be depolymerized in living organisms [70]. According to literature studies, eight human chitinases involved in different complexes can degrade this polymer. Their enzymatic activity acts on the chitosan backbone and breaks the chemical bonds, resulting in non-toxic residues of different lengths. The results, known as oligosaccharides, are further integrated into metabolic processes and are eliminated without inducing damage in tissues. The first factor involved in this degradation is related to the DD, and the second factor involved is associated with the arrangement of chemical bonds in the molecules [71,72]. The main degradation residues are N-acetyl glucose and glucosamine, which are harmful to human tissues. Degradation intermediates obtained from reactions do not agglomerate, so they are eliminated in time [71]. Poth et al. [73] tested nanoparticles based on chitosan-tripolyphosphate (TPP) in vitro in lysozyme solution (1.5 μg/mL) for faster osseointegration of endoprostheses after surgical interventions. For chitosan, two DDs were selected: 17% and 42%. In the case of chitosan with higher DD, nanoparticles degradation was achieved in 4 days, while for 17% DD, size reduction did not occur in 7 days. Even if the enzyme solution concentration was increased, the results remained the same [73]. The action of lysozyme solution (2 mg/mL) was also observed by El-Sherviny et al. [74], which revealed the degradation of a hydrogel based on chitosan/polyethylene glycol and Pluronic F-108 as a solution for pulmonary drug delivery. The biodegradation began very fast, within the first hour, and just after 6 h, half of the hydrogel mass was already reduced [74].

### 3.3. Antimicrobial Activity

**Figure 5:**
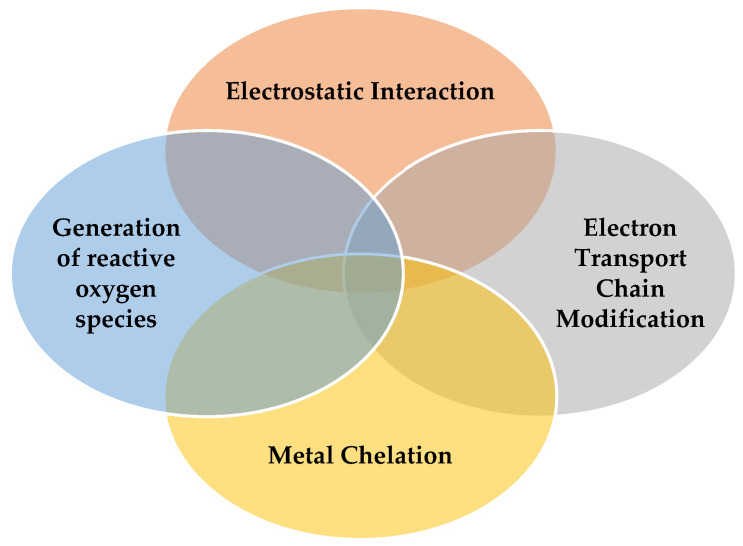
Main factors involved in the antibacterial activity of chitosan-based materials.

Chitosan and their derivatives exhibit varying efficacy against Gram-negative and Gram-positive bacteria. The antimicrobial effect is related to the interactions between polycationic chitosan and the anionic chemical groups from the cell membrane. This interaction allows the disintegration of the cell and the leakage of various intracellular constituents [75,76]. The mechanism is complex and depends on several factors, summarized below in Figure 5.

- *Electrostatic Interaction*:

The most widely accepted mechanism involves electrostatic interactions between the positively charged amino groups of chitosan and the negatively charged cell membranes of bacteria. This interaction disrupts membrane permeability, leading to osmotic imbalance, efflux of intracellular substances, and, ultimately, cell death [76,77,78,79].

- *Electron Transport Chain Modification*:

Chitosan can also modify the bacterial electron transport chain [80,81]. Chitosan with low molecular weight can enter the cell and directly inhibit DNA replication [80].

- *Metal Chelation*:

Chitosan’s chelating capacity for metal ions can stimulate toxin production, thereby affecting bacterial viability [82]. This is because metal ions bound to the bacterial cell wall are crucial for its stability, and their removal by chelation disrupts vital functions. This chelating mechanism is more effective at high pH due to the availability of electron pairs on amine groups [83].

- *Generation of reactive oxygen species*:

The antimicrobial activity is attributed to the generation of reactive oxygen species (•OH, H_2_O_2_, and O_2_^2−^) by these nanomaterials, which damage microbial cells, indicating their potential use in biomedical antibacterial applications [84]. Chitosan polycationic structure attaches to the negatively charged cell membranes, leading to an instability in the osmosis of the cell. This interaction leads further to a leakage of intracellular constituents, thus promoting the appearance of reactive oxygen species and affecting cells’ normal functioning [85].

The antibacterial action of chitosan can be viewed as several successive steps. First, the polycationic charge of the chitosan molecule facilitates strong binding to the microbial cell surface, which causes the cell membrane to gradually shrink and ultimately leads to cell death. Then, polycationic chitosan molecules can interact with primarily anionic components of the cell wall, such as lipopolysaccharides and proteins, resulting in the leakage of intracellular components due to alterations in the permeability barrier, which, in turn, inhibits nutrient transport across the cell membrane. After that, chitosan, particularly in its low-molecular-weight or nanoparticle forms, can infiltrate the cell, bind to DNA (a polyanion), and subsequently hinder RNA and protein synthesis. Finally, polycationic chitosan can induce cell coagulation, leading to their precipitation [86,87].

Antibacterial activity is influenced by various factors that can control the final properties of chitosan materials, as can be observed in Table 1. The antibacterial effect of chitosan can be increased by the use of various metallic nanoparticles known for their antimicrobial effect [15,88]. Further, some possible factors are presented that affect the antibacterial effectiveness of chitosan-based materials.

## 4. Applications

### 4.1. Skin and Bone Tissue Engineering

**Figure 6:**
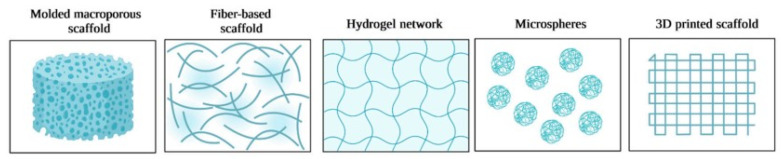
Three-dimensional scaffolds based on chitosan as biomaterials for tissue engineering. Reprinted from an open-source article [47].

Tissue engineering has been used in recent years for the reconstruction of tissues and organs injured or for the replacement of specific damaged tissue in living organisms. The development of the medical engineering field has allowed for the fabrication of alternative materials able to simulate the original extracellular matrix of the body by maintaining or improving tissue functions [107,108]. The design of these types of similarities between the material and the extracellular matrix can offer better interaction between human cells and the biomaterial and can induce faster tissue regeneration. In tissue engineering, the cells that support the healing processes in the body are activated through growth factors or genes, so they initiate the reconstruction of the new targeted tissue [109]. Hydrogels and scaffolds based on chitosan are among the most developed materials for skin/bone tissue engineering. Examples of three-dimensional scaffolds are presented below in Figure 6, according to a recent open-access review article [47].

Tissue engineering includes the study of engineering and materials science in order to improve the patient’s quality of life and to create innovative treatments for medical diseases. Biomaterials based on natural polymers, such as chitosan, can be considered an adequate solution to repair or support injured tissue for better evolution [110]. In the last few years, the development of hydrogels and scaffolds has increased in medical areas due to their good adaptability in the body. Advantages such as faster recovery, less use of transplants, smaller number of donors, non-toxicity, and reduction in the number of animals used in biological experiments are reasons for which chitosan-based polymeric materials are intensively studied. Due to their geometric shape and high porosity, they can replace the damaged tissue by accelerating healing and supporting the recovery of damaged organs by encouraging cell sedimentation [111,112]. Hydrogels are biomaterials organized in the form of a three-dimensional network of polymer chains [113] used mostly in tissue engineering applications due to their superior properties compared to other dressings: biocompatibility, biodegradability, elasticity, porosity, water uptake, and good swelling properties in exudate absorption from wounds [114,115]. Hydrogels are 3D materials with a high percentage of water and solvents in their structure [116]. They can be easily manipulated in many forms and are comfortable and versatile depending on their destination [117]. They are capable of inducing modifications in volume depending on the pH of the medium, local absorption, or exterior stimuli applied [118]. The properties of these forms are controllable and superior to the solutions from which they are obtained [119]. Hydrogels can be hydrated by water or biological fluids.

The classification of hydrogels can be made based on the polymers used or their internal structure [120]. Hydrogels are described as two types, depending on their structure [121,122]. First, there are the chemical gels (permanent gels), which are covalently crosslinked through covalent bonds. They are strong hydrogels, able to offer great support, and they can offer an equilibrium when swelling is happening [123]. Second, there are the physical gels (reversible gels), which are based on weaker bonds. The polymeric network is held together through ionic or hydrogen interactions. In physical gels, the interactions between chains are weaker compared with chemical hydrogels. The bonds are reversible, and they can be broken in different reaction conditions [124,125,126]. Chitosan presents an innovative property in relation to other polymers since it can create a gel network by itself without the addition of reticular agents. Of course, for other applications where a stronger material is needed, reticular agents could be introduced into the synthesis. However, in the case of medical applications, it is desirable to use as few chemical reagents as possible since these devices come in direct contact with the skin. The ability of chitosan to form a hydrogel is due to the chemical neutralization of positive amino groups in its backbone and to the apparent repulsions between chains [71].

Furthermore, chitosan can create complex structures in hydrogels with other natural or synthetic polymers. Polyvinyl alcohol (PVA) or polyethylene glycol (PEG) bonded to chitosan via crosslinking are just some examples [127]. In the case of natural polymers, the network bonds are obtained through negatively charged molecules. Collagen and gelatine are examples of natural polymers used to improve chitosan’s final properties [71]. Since chitosan is a natural polymer, its mechanical properties could be reinforced using various other reagents for specific applications. Table 2, inspired by [24], presents examples of complexes formed by chitosan composite materials.

A hydrogel based on gelatine–chitosan and PVA was developed by Rodríguez-Rodríguez et al. [141]. The composite proved to have good chemical and mechanical properties due to the synthetic polymer, while the swelling behavior and the cytotoxicity showed some positive results. The hydrogel based on chitosan revealed optimum pore sizes for tissue engineering so that the attachment of cells and the diffusion of nutrients was possible. For biocompatibility testing, the scaffold was tested on HT-29 cells. The cell viability did not decrease significantly after many days, so the results encouraged its use for longer treatment without damaging the tissues [141].

Besides soft tissue engineering, chitosan is also used in bone and cartilage regeneration. Hydroxyapatite is a material that can reinforce chitosan’s mechanical properties due to its similarities to bone components and minerals. A recent work showed that lyophilized chitosan/hydroxyapatite scaffold can modulate osteoblast maturation depending on the size of the particles. Smaller sizes of hydroxyapatite particles seem to improve the maturation process and decrease the inflammatory state [142]. Besides hydroxyapatite for bone reconstruction, there are also other materials used together with chitosan, such as zirconia, nano calcium zirconate, and metal alloys, with remarkable mechanical properties. In a recent study, a composite scaffold based on chitosan and chondroitin sulfate with nano-sized bioglass in its structure was developed. The in vitro analysis of osteoblast cells showed an increase in bioactivity regarding apatite deposition and type 1 collagen expression [143]. The overview published by Signorini et al. [144] reported the use of chitosan-based materials as scaffolds for bone regeneration in dentistry since they promote cell adhesion and proliferation, increase vascularization and mineralization, and create a favorable medium for bone rejuvenation by sustaining calcium phosphate expression [144].

### 4.2. Wound Dressing

Chitosan is also used in the healing process as wound dressing material. To act as a dressing, biocompatibility, non-toxicity, and the ability to provide a moist environment must be fulfilled by the proposed material. Wound dressings are usually formulated as films, gels, fibers, spheres, or membranes. Other aspects are related to the antibacterial effect, which should also be considered since infections commonly occur at the wound area. The chitosan-based wound dressing should be able to offer a proper temperature for re-epithelization and collagen deposition and should also be comfortable on the skin to avoid traumatic removal after the regeneration process [55].

Zhang et al. [145] proposed a carboxyl-modified PVA/chitosan thin film synthesized by crosslinking using an amide linkage formation as a wound dressing. Due to PVA, the final mechanical properties were improved, while the biocompatibility of the dressing remained unchanged. The obtained wound dressing was able to maintain a humid area over the wound in order to facilitate healing [145]. Patil et al. [146] developed a moist wound dressing for diabetic wounds based on fluorinated methacrylamide chitosan designed to enhance oxygenation and increase the healing process. Since hypoxia is the key factor for the inflammation process, the dressing aims to promote collagen synthesis, angiogenesis, and re-epithelialization by providing local oxygenation and collagen deposition. Chitosan served as a biological support for the healing process to occur [146]. In a recent study, Wu et al. [147] fabricated microspheres based on chitosan and calcium phosphate in an aqueous solution using a hydrothermal method and various annealing temperatures (150–700°) for bone infection treatment. The physico-chemical characterization performed by XRD, FT-IR, TEM, and SEM revealed the formation of composite spheres with pores assembled by petal-like flakes. The influence of the temperature was studied since chitosan was used as a chelating agent. After annealing at 150 °C, the petal-like flake forms collapsed in narrow plates. Next, at 300 °C, the degradation of chitosan occurred. At the highest temperature, 700 °C, the morphology of the plates was transformed into round particles, while chitosan was completely combusted. The study revealed the method as a successful one to obtain the phase transformation from hydroxyapatite and calcium phosphate to β-TCP [147].

### 4.3. Drug Delivery

**Figure 7:**
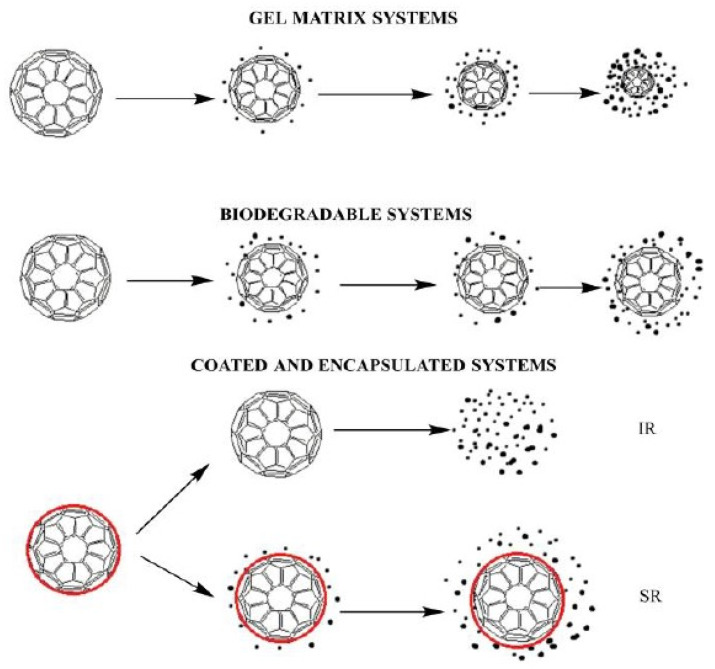
Mechanisms of degradation for chitosan-based drug delivery blends: gel matrix systems/biodegradable systems (the drug is homogeneously distributed in a gel polymer matrix/in a biodegradable system) or coated/encapsulated systems (the drug is covered by a polymeric film that dissolves quickly in coated immediate-release (IR) systems, while the drug release gradually occurs in coated sustained-release (SR) systems due to the diffusion process). Reprinted from an open-source article [150].

Chitosan is used in drug delivery systems since it is biodegradable and bioactive, it can increase the retention time of the drugs, it does not show any side effects, and it can be controlled to obtain a specific porosity or form [148,149]. Drug delivery can be approached through different mechanisms, depending on the targeted area or the time release needed. In a recent review paper, Kaur et al. [150] described, as can be observed in Figure 7, various systems in which the polymer can be degraded and the drug released in further biomedical applications.

In the first and second cases, when a gel matrix system is fabricated, the polymer is usually mixed with the drug into a biodegradable system. Biodegradation occurs depending on the mechanical properties of the polymer used. For chitosan, an addition of synthetic polymer could be a solution for a stronger system. For instance, its association with PVA increases its mechanical strength, making it suitable for articular applications [151]. The last system is based on the coating and encapsulation of the drug into a polymeric matrix through which the drug can diffuse. The encapsulated drug is released slowly while the polymeric film dissolves. The two approaches described are based on the release time. Either the drug is immediately released from the polymeric matrix (IR—immediate-release), or it can be slowly released over a longer time (SR—sustained-release) [150]. Different research studies confirm that chitosan is suitable for encapsulating various biomolecules, drugs, or bioactive compounds. Chen et al. [152] proposed an innovative co-delivery system in which chitosan-coated zein nanoparticles were used as a matrix for both curcumin and resveratrol compounds. The center of the nanoparticles was designed for curcumin, while resveratrol was absorbed on the surface of the nanocomplexes that were created. The storage stability tests showed better retention of curcumin when a low MW chitosan was used. The retention percentages obtained were 85% for low MW chitosan, 81.1% for medium MW chitosan, and 78.4% for high MW chitosan, suggesting that the system can successfully protect the drugs from external stresses on long-term storage [152].

In the case of drug delivery, some factors can influence the encapsulation and release rate of agents. The stability of chitosan interaction with therapeutic agents depends on the molecular weight, temperature, pH, and surface charge of the drug. To increase tissue healing, nanoparticles or drugs can be introduced into the reaction [24]. Some applications are mentioned in Table 3.

### 4.4. Antibacterial Applications

Chitosan acts as a broad-spectrum antibacterial agent, particularly when combined with other materials like graphene oxide or zinc oxide that enhance its effectiveness in different processes, including biomedical or water treatment. Chitosan’s ability to inhibit bacterial growth has been widely researched during the last few years. This makes it a valuable component for antibacterial membranes, particularly in wastewater treatment, where membrane fouling by bacteria can reduce efficiency.

The antibacterial properties of zinc oxide nanoparticles combined with chitosan were revealed in several studies. In one of them, bentonite-supported silver and zinc oxide nanoparticles in a chitosan matrix were used to disinfect water, showing excellent antibacterial effects against *E. coli* [100]. Another work found that zinc oxide–chitosan coatings applied on microfibrillar cellulose also displayed strong antibacterial activity, again tested against *E. coli*, further proving the efficiency of chitosan nanocomposites in bacterial inhibition [164]. Another study on magnetic chitosan/graphene oxide nanocomposites found these materials to have enhanced antibacterial activity, particularly against *E. coli*. This effect is attributed to damage caused to the bacterial cell membrane, demonstrating the efficacy of chitosan/graphene oxide composites in antibacterial applications [165].

The work presented by Jamshidi et al. [84] reported on synthesized novel zinc mesoporous silica nanoparticles and a biocomposite using UV radiation, combining zinc mesoporous silica nanoparticles with an organic matrix of chitosan and graphite. Both nanomaterials demonstrated effective antibacterial activity against *S. aureus* and *E. coli* across concentrations of 5, 10, and 100 µg/mL after an 18 h exposure at 310 K [84]. The bacteriostatic and fungistatic qualities of chitosan-based materials are also advantageous for wound treatment. Beyond their antimicrobial properties, chitosan and its oligosaccharides can promote cell growth. Chitosan-based materials, such as non-wovens, nanofibers, composites, films, and sponges, have been demonstrated to aid wound healing and dermal regeneration, making wound healing the primary commercial application of chitosan in the biomedical field [166]. Guarnieri et al. [167] reported on the antimicrobial potential of chitosan from various *Hermetia illucens* (*H. illucens*) biomasses, including larvae, pupal exuviae, and adult remains. All tested samples demonstrated inhibition against both *E. coli* and *Mastacembelus favus* (*M. favus*) strains, with strong antimicrobial effects confirmed through microdilution assays. The study reports that both bleached and unbleached pupal exuviae exhibited significant antibacterial activity, underscoring the potential of *H. illucens* as a viable alternative source of chitosan for biomedical and pharmaceutical applications. The work described by Mohammed et al. [168] describes the comparison between a hydrogel/cryogel microspheres based on low MW chitosan and green synthesized silver nanoparticles and a commercial antibiotic as an antimicrobial agent. The study proved that the system based on 7% wt chitosan and various concentrations of silver nanoparticles can successfully inhibit different bacteria strains, among them *S. aureus*, *Proteus vulgaris* (*P. vulgaris*), *E. coli*, and *Pseudomonas aeruginosa* (*P. aeruginosa*). Using the disk diffusion method, the results suggested that when the silver nanoparticle concentration is higher, the antibacterial effect is also stronger, with the highest inhibition at 2% wt [168]. The study presented by Hermosilla et al. [169] shows chitosan as a coating for *Stereum hirsutum* fungus used further in the green synthesis of nanoparticles. The results show a stable complex that can be used multiple times for nanoparticle synthesis and can offer an antibacterial effect in the final material [169].

### 4.5. Bioprinting

Bioprinting is an additive manufacturing technique used to create three-dimensional structures for biomedical applications. It involves the deposition of biomaterials, including cells, hydrogels, and growth factors, in a layered manner to create functional tissues. The aim is to generate tissues for transplantation, drug screening, or disease modeling.

Chitosan is a valuable biomaterial in bioprinting because of its biocompatibility. Numerous studies like [170,171], have demonstrated the biocompatibility of chitosan, showing minimal inflammatory responses. Moreover, chitosan is biodegradable. Chitosan’s degradation rate can be controlled through modifications, making it suitable for temporary scaffolds [172]. Chitosan’s mechanical properties make this polymer attractive for bioprinting. Its mechanical properties are adjustable through modifications [173] and allow it to mimic the stiffness of various tissues. Chitosan presents an inherent antimicrobial activity, which contributes to infection prevention, which was treated in detail in a previous subsection. Moreover, due to its positive charge, it promotes cell attachment and proliferation [174]. Due to these particular properties, chitosan has emerged as a versatile material in the field of bioprinting, particularly for the development of biosensors. Its unique properties, such as film-forming ability, biocompatibility, and biodegradability, make it an excellent candidate for various biomedical applications.

Chitosan’s ability to form films is crucial for biosensor applications. These films can be easily modified to incorporate various sensing elements, enhancing the sensor’s functionality. The biocompatibility of chitosan ensures that it does not elicit adverse immune responses when used in biological environments, making it suitable for in vivo applications [175]. Some of the biomedical applications are mentioned below.

Chitosan-based biosensors are widely used for glucose monitoring. The film-forming ability of chitosan allows for the creation of thin, uniform layers that can immobilize enzymes such as glucose oxidase. This enzyme catalyzes the oxidation of glucose, producing a measurable signal that correlates with glucose concentration [176,177]. These sensors are particularly useful for managing diabetes, as they provide real-time glucose levels with high accuracy. Chitosan is an excellent material for biosensors aimed at detecting pathogens. Chitosan films can be functionalized with specific antibodies or nucleic acids that bind to target pathogens. Upon binding, these sensors produce a detectable signal, enabling rapid and accurate pathogen detection [178,179]. This application is critical in clinical diagnostics and food safety. Chitosan is also used in the fabrication of electrochemical biosensors. Its film-forming ability allows for the creation of conductive films that can be used to detect various analytes. For instance, chitosan-based electrochemical sensors have been developed for detecting heavy metals, toxins, and other environmental pollutants [180,181,182,183]. These sensors offer high sensitivity and selectivity, making them valuable tools for environmental monitoring.

The flexibility and biocompatibility of chitosan also make it suitable for wearable biosensors. These sensors can be integrated into wearable devices to continuously monitor physiological parameters such as heart rate, body temperature, and sweat composition. The use of chitosan ensures that these devices are comfortable to wear and do not cause skin irritation.

The use of chitosan in bioprinting biosensors offers several advantages. Firstly, chitosan is naturally biodegradable, reducing the environmental impact of disposable biosensors. Another advantage derives from the fact that chitosan films can be easily functionalized with various biomolecules, enhancing the specificity and sensitivity of biosensors. Moreover, chitosan is relatively inexpensive compared to other biopolymers, making it an attractive option for the large-scale production of biosensors. Future research is likely to focus on enhancing the performance of chitosan-based biosensors through the incorporation of nanomaterials and advanced fabrication techniques [14,184]. These advancements could lead to the development of highly sensitive, multi-functional biosensors for a wide range of biomedical applications.

### 4.6. Food Industry

Chitosan has gained significant attention in the food industry due to its unique properties and versatile applications. Chitosan exhibits strong antimicrobial activity against a wide range of foodborne pathogens, including bacteria, yeasts, and molds [78]. This property makes it an excellent natural preservative for extending the shelf life of various food products. Another application in the food industry is due to the fact that chitosan can form transparent, biodegradable films and coatings that can be used to protect fresh fruits, vegetables, and other perishable foods. Chitosan-based edible coatings and films have shown remarkable potential in extending the shelf life of fresh produce, meats, and seafood. These coatings help reduce moisture loss, control gas exchange, and prevent microbial growth [185,186].

Chitosan is used in the beverage industry for clarifying and deacidifying fruit juices and wines. It can effectively remove suspended particles, proteins, and phenolics, resulting in clearer and more stable products [187]. Its ability to form complexes with proteins, polyphenols, and other suspended particles makes it an effective fining agent [188]. Many food products, such as sauces, dressings, and mayonnaise, consist mainly of oil-in-water emulsions. Chitosan’s emulsifying properties make it useful for stabilizing oil-in-water emulsions, as revealed by many studies [189,190,191]. Other studies revealed the use of chitosan to encapsulate and protect sensitive bioactive compounds, such as vitamins, antioxidants, and probiotics, improving their stability and bioavailability in food products [192,193,194,195].

Chitosan’s ability to bind dietary fats has led to its use in developing reduced-fat food products. It can help create a creamy texture and mouthfeel in low-fat alternatives to traditional high-fat foods [196]. The study in [197] highlights the potential of edible chitosan films as a promising material for food packaging, with specific recommendations for future improvements to enhance their properties. Chitosan is also considered a dietary fiber and has been investigated for its potential health benefits, including cholesterol-lowering effects and weight management properties, as revealed by [198].

### 4.7. Water Treatment

Chitosan and its derivatives have emerged as powerful tools for water purification and wastewater treatment, demonstrating exceptional capabilities in removing a wide range of pollutants. These versatile biopolymers have been extensively studied for their ability to tackle various contaminants, including heavy metals, dyes, and other organic pollutants.

Innovative materials like wastepaper/chitosan aerogels have shown enhanced copper adsorption capabilities with a capacity of 156.3 mg/g, surpassing that of chitosan alone [199]. For nickel removal, carboxylate-rich magnetic chitosan flocculants have demonstrated an impressive uptake rate of 98.3% for Ni(II) within just 60 min at pH 4–8 [200]. Chitosan cross-linked with amino acids, such as glutamic acid, has demonstrated excellent lead removal, with a maximum capacity of 91% at pH 4 [201]. Even in challenging environments like seawater, chitosan-based materials have proven effective, with a poly(amidoxime)-grafted chitosan/bentonite composite showing strong adsorption of U(VI) from seawater, reaching a maximum adsorption capacity for uranium of 49.09 mg/g at pH 8 [202]. Montmorillonite–chitosan composites have shown promise in removing lead, copper, and cadmium from water, with adsorption kinetics following pseudo-second-order models [203].

In the quest for metal ion removal, chitosan-based materials have shown remarkable effectiveness. For instance, magnetic β-cyclodextrin-chitosan/graphene oxide composites have demonstrated superior adsorption capacity for Cr(VI) at 67.66 mg/g, outperforming many other sorbents due to their large surface area and the presence of hydroxyl and amino groups [204]. Similarly, graphene oxide-functionalized chitosan has proven highly effective in arsenic removal, with adsorption capacities of 64.2 mg/g for As(III) and 71.9 mg/g for As(V) at an optimal pH range of 4.3–6.5 [205].

When it comes to organic pollutants, particularly dyes, chitosan-based materials continue to impress. Xanthate-modified magnetic chitosan demonstrated high adsorption capacities of 197.8 mg/g for methylene blue and 169.8 mg/g for safranin O at 35 °C [206]. Chitosan biocomposite adsorbents have achieved impressive adsorption capacities of 362 mg/g and 398 mg/g for safranine T and brilliant cresyl, respectively [207]. These materials have also shown versatility in removing mixed dyes, such as cationic methyl violet and anionic alizarin yellow R, with adsorption kinetics following pseudo-second-order equations [208]. Magnetic chitosan microspheres surface-grafted with poly(2-(dimethylamino)ethyl methacrylate) have shown high efficiency in removing anionic dyes like acid green 25 and reactive blue 19, retaining high performance even after multiple adsorption–desorption cycles [209]. Chitosan-based nanocomposite films incorporating polyvinyl alcohol and ZnO nanoparticles have been developed for acid black removal, offering improved adsorption performance and reduced toxicity compared to pure chitosan/PVA films [210]. In another study, a chitosan solution achieved remarkable adsorption capacities of 450 mg/g for Cu(II) and 384 mg/g for Ni(II) at pH 5.2, significantly higher than previously reported capacities for raw chitosan [211]. Beyond dyes, chitosan/graphene oxide nanocomposite fibers templated with copper nanoparticles have demonstrated excellent capabilities in detecting and reducing 4-nitrophenol from polluted water [212].

These diverse applications highlight the immense potential of chitosan-based materials in addressing a wide spectrum of water pollution challenges, making them invaluable assets in the field of environmental remediation and water treatment.

## 5. Conclusions

Chitosan is a natural biopolymer with remarkable biocompatibility and biodegradability properties used in various types of applications. Chitosan’s abundance in nature and its wound-healing properties make it a valuable biopolymer in regenerative medicine. Soluble in acidic solution and able to be chemically modified, chitosan can be successfully obtained in different chemical derivatives with superior properties for further biomedical or technological purposes. As an innovative biomaterial, chitosan is involved in composite systems to offer support for future tissue regeneration, drug delivery systems, and antibacterial applications, as well as the food packaging and water treatment fields. Continuing research in tissue engineering has led to the development of smart hydrogels that can be activated in specific conditions for medical purposes. This review highlights chitosan’s properties related to the biomedical field, with a focus on the antibacterial activity of different chitosan composite materials. Its antimicrobial activity depends on many factors, such as pH, concentration, molecular weight, and degree of acetylation.

The main focus of the review is related to chitosan applications in biomedical applications, the food industry, and water treatment. Several recent studies have been addressed to show the efficiency of chitosan in skin and bone hydrogels and scaffolds, wound dressings, drug delivery systems, and antimicrobial composites. Its ability to offer support for cell sedimentation and to deliver biocompounds to a target organ has led to a deeper investigation in the science literature regarding chitosan/other polymers or chitosan/metallic nanoparticle materials. Lately, the food industry and environmental organizations have also proposed chitosan as a biodegradable material for packaging since it is non-toxic polymer with naturally degradable compounds.

## Figures and Tables

**Table 1:** Factors involved in antimicrobial activity of chitosan.

Factor	Antibacterial Activity
**Bacterial Species** **(Gram-negative vs. Gram-positive)**	While some studies show greater activity against Gram-negative bacteria [82,89,90], others report higher sensitivity in Gram-positive bacteria [91]. This difference is partly attributed to the variations in cell wall and membrane composition. Gram-negative bacteria have an outer membrane with lipopolysaccharide, which contains anionic groups, making the surface more negatively charged than Gram-positive bacteria, which have a cell wall made of peptidoglycan and teichoic acid [92,93,94,95]. Studies have demonstrated varied results depending on the specific bacteria being tested.
**Growth Stage**	Bacterial growth stage affects susceptibility to chitosan materials. Mid-exponential growth phase demonstrates higher susceptibility than the late-exponential phase [96]. Changes in cell surface negativity during different growth stages also contribute to the difference in susceptibility [97].
**Zeta Potential**	Higher zeta potential (positive charge) generally correlates with increased antibacterial activity [98,99]. This positive charge improves electrostatic interactions with the negatively charged bacterial cells. Metal ion incorporation influences zeta potential, as positively charged ions increase it while negatively charged ones decrease it [98].
**Concentration**	Higher concentrations generally lead to enhanced antibacterial effects [100,101,102]. For instance, for chitosan nanoparticles, the optimal concentration varies depending on factors such as bacterial species and nanoparticles’ characteristics after the synthesis.
**pH**	Antibacterial activity often depends on pH. Acidic conditions generally enhance activity due to the protonation of the –NH_2_ groups, increasing the positive charge and improving interaction with bacterial cells [103]. However, this is not consistent across all studies and may vary by bacterial species.
**Molecular Weight and Degree of Acetylation**	Lower MW chitosan materials generally exhibit higher antibacterial activity due to higher zeta potential and smaller particle sizes [91,104,105]. The DD is also important. Studies show that higher DD often correlates with increased positive charge and thus enhanced antibacterial activity [106].

**Table 2:** Chitosan composites with biomedical applications.

Composite	Application	References
**Chitosan** **–** **alginate**	Hydrogel pores for new tissue regeneration	[128]
**Chitosan** **–** **collagen**	Regeneration of skin/organ and vascularization improvement	[129]
**Chitosan** **–** **collagen**	Corneal nerves, stroma and epithelium reconstruction	[130]
**Chitosan** **–** **gelatine**	Scaffold and gel applications for further cell proliferation due to gelatine presence	[131]
**Chitosan** **–** **bacterial cellulose**	Bacteriostatic activity improved as chitosan concentration increases	[132]
**Chitosan** **–** **pectin**	Adhesion and differentiation of human osteoblast cells	[133]
**Methacrylate glycol chitosan** **–** **hyaluronic acid**	New cartilaginous extracellular matrix formation	[134]
**Chitosan** **–** **Au**	Increased cardiomyogenic cell differentiation	[135]
**Chitosan** **–** **sodium hyaluronate with bioglasses**	Bone regeneration	[136]
**Chitosan** **–** **sodium bicarbonate**	Biocompatible porous hydrogel proposed as scaffold	[137]
**Chitosan** **–** **cordycepin**	Hydrogel with antibacterial and antioxidant effects	[138]
**Chitosan** **–** **graphene oxide**	Smart hydrogel with improved mechanical properties for biocompounds encapsulation	[139]
**Chitosan with zwitterionic sulfopropylbetaine and poly(2-hydroxyethyl acrylate)**	Hydrogels for skin tissue engineering with good mechanical and antibacterial properties	[140]

**Table 3:** Drug delivery systems based on chitosan and composites.

Material	Drug	System Developed	References
**Chitosan–alginate**	BSA	Smart pH-sensitive system for BSA release at gastric fluid level	[153]
**Chitosan–alginate**	Insulin	Improved hydrogel for encapsulation at neutral pH	[154]
**Chitosan–PVA**	Insulin	Thermal innovative gel for slow release of insulin	[155]
**Chitosan–PVA–hydroxyapatite**	BSA	Improved hydrogel for BSA protection for a longer time	[156]
**Chitosan–Au**	Doxorubicin	Improved encapsulation efficiency since a high percentage of the drug was released after a long time under physiological conditions	[157]
**Chitosan–folate with magnetic nanoparticles**	Doxorubicin	Smart hydrogel pH-sensitive for anticancer activity	[158]
**Chitosan–montmorillonite**	Famotidine	Mucoadhesive bio-nanocomposite as a gastroretentive carrier to prolong retaining the drug in the stomach	[159]
**Chitosan–magnetic nanoparticles and Mg-Al double-layered hydroxides**	Diclofenac	Smart pH-sensitive drug system for anti-inflammatory effect	[160]
**Chitosan–bacterial cellulose**	Naproxen	Smart pH system for clinical therapy of digestive issues	[161]
**Chitosan–carboxymethylcellulose**	Cannabidiol	Injectable hydrogel for spinal cord injury	[162]
**Chitosan–polycaprolactone**	Capsaicin	Nanoplatform for anticancer activity that can inhibit the proliferation of MCF-7 human breast cells, while the dermal fibroblasts did not show any toxicity	[163]
